# Atypical Melanoma of the Lateral Fifth Metatarsal Shaft: A Rare Presentation With Minimal Mitotic Activity

**DOI:** 10.7759/cureus.106743

**Published:** 2026-04-09

**Authors:** Samantha Reed, Joseph Torkieh, Ruthesh Udayaraj, Zoya Tariq, Jodene Shwer, Rahul Mittal

**Affiliations:** 1 Podiatry, Cooperman Barnabas Medical Center, Livingston, USA; 2 Medicine, Rutgers Robert Wood Johnson Medical School, New Brunswick, USA; 3 Health Informatics, Rutgers University, Piscataway, USA; 4 Pathology, Cooperman Barnabas Medical Center, Livingston, USA

**Keywords:** amelanotic melanoma, foot melanoma, low mitotic index, not otherwise specified melanoma, soft-tissue mass

## Abstract

Melanoma of the foot is an uncommon malignancy that often presents with atypical clinical features, contributing to delayed diagnosis and poorer outcomes. We report a rare case of an 88-year-old female with a minimally mitotic, amelanotic melanoma arising from the lateral fifth metatarsal shaft, initially concealed beneath a callus. The lesion was painless in its early stages and not clinically apparent, resulting in a diagnostic delay of more than three years. Magnetic resonance imaging (MRI) ultimately revealed a pedunculated soft-tissue mass suspicious for malignancy. Given the presence of significant peripheral arterial disease, revascularization was performed prior to definitive surgical management. The patient subsequently underwent complete surgical excision of the lesion. Histopathological analysis confirmed melanoma classified as Not Otherwise Specified (NOS), a category used when tumors do not fit established histopathologic subtypes.

This case highlights the diagnostic challenges associated with amelanotic and atypically located melanomas of the foot and underscores the importance of careful evaluation of persistent or unusual foot lesions. It emphasizes the need for heightened clinical suspicion in elderly and diabetic populations to facilitate earlier diagnosis and improved outcomes.

## Introduction

Melanoma is a malignant neoplasm arising from melanocytes and represents one of the most aggressive forms of cutaneous malignancy [1,2]. Globally, approximately 330,000 new cases were diagnosed in 2022, with incidence continuing to rise [3]. In the United States, projections for 2025 estimate approximately 104,960 new cases and over 8,400 melanoma-related deaths [4]. Although ultraviolet (UV) radiation exposure is the predominant risk factor in fair-skinned populations, melanoma may also arise in non-sun-exposed regions, particularly in individuals with darker skin types [1,5]. Acral sites, including the palms, soles, and subungual regions, are disproportionately affected in these populations [6]. Foot melanomas are relatively uncommon overall, accounting for approximately 2-3% of all cutaneous melanomas, but they are frequently diagnosed at more advanced stages due to delayed recognition [7]. Lesions involving the plantar surface or lateral foot may mimic benign conditions, such as calluses, warts, or traumatic injuries, thereby contributing to significant diagnostic delay [8].

The major histopathologic subtypes of melanoma include superficial spreading melanoma, nodular melanoma, lentigo maligna melanoma, and acral lentiginous melanoma [6]. Less common variants, including amelanotic melanoma and melanomas classified as Not Otherwise Specified (NOS), may lack classic clinical and morphologic features, further complicating diagnosis [2]. In such cases, immunohistochemistry is essential for accurate diagnosis. Melanocytic markers, such as S100 and SOX10, demonstrate high sensitivity and aid in confirming melanocytic origin, particularly in poorly differentiated or amelanotic tumors, while proliferation markers, such as Ki-67, provide additional information regarding tumor activity and aggressiveness [2,7].

We present a rare case of minimally mitotic, amelanotic melanoma arising from the lateral aspect of the fifth metatarsal shaft, initially concealed beneath a callus. This case highlights the diagnostic challenges associated with atypical acral melanoma presentations and underscores the importance of maintaining a high index of suspicion for malignancy in persistent or unusual foot lesions.

## Case presentation

An 88-year-old African American female with a past medical history significant for hypertension, hyperlipidemia, diabetes mellitus, and coronary artery disease presented to the emergency department with complaints of an 8/10 painful mass on the lateral aspect of her left foot. The pain was unresponsive to over-the-counter topical analgesics. She was diagnosed with a soft-tissue mass of unknown etiology and advised to follow up with podiatry within one week, as there was no immediate concern for metastasis or need for emergent intervention.

At that time, the assessment was based on clinical evaluation alone, with no imaging or biopsy performed. The lesion was described as a localized soft-tissue swelling without overt features of malignancy, contributing to the initial conservative management approach. She was instructed to manage symptoms with acetaminophen and nonsteroidal anti-inflammatory drugs (NSAIDs). The patient was subsequently lost to follow-up for approximately three years.

**Figure 1:**
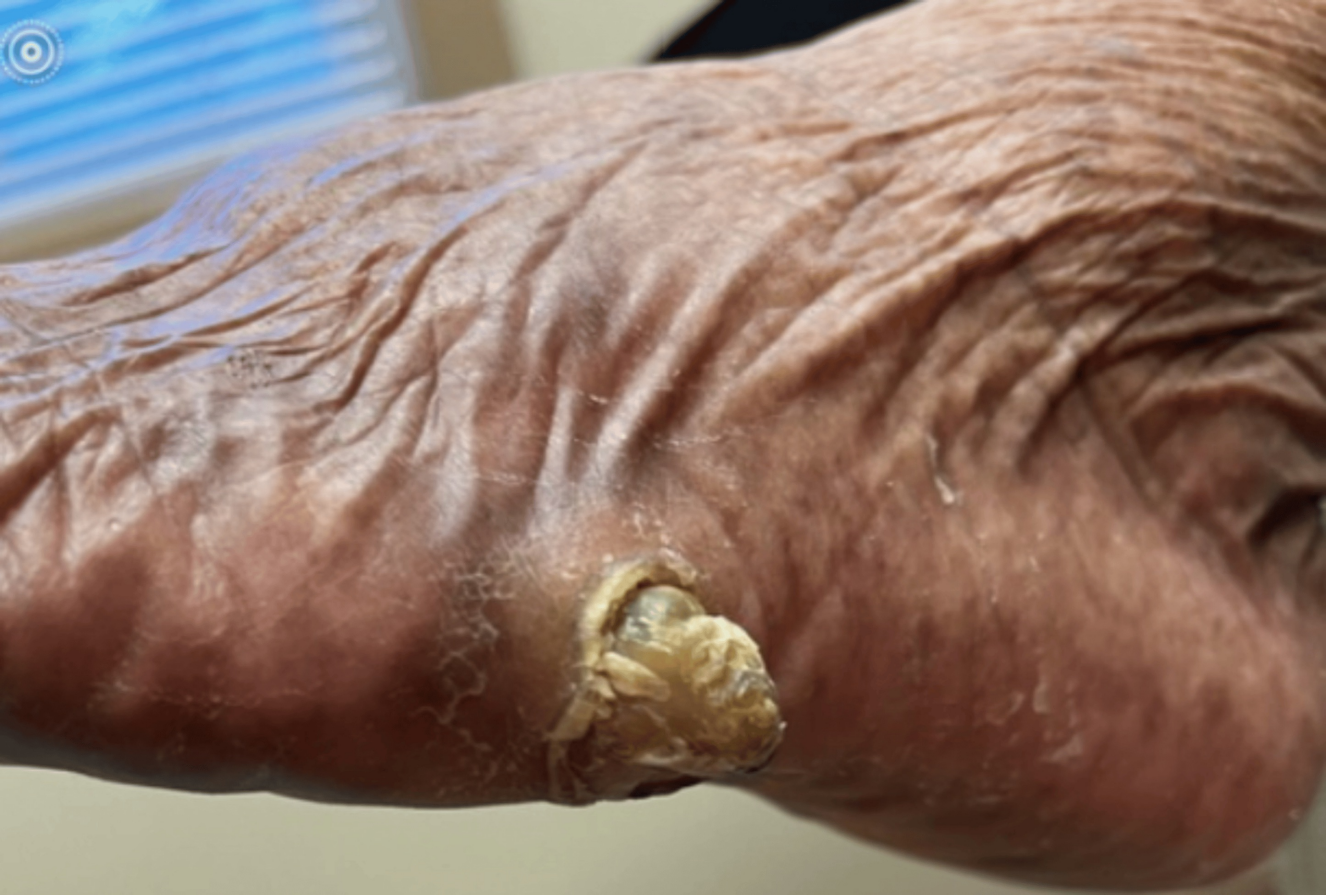
Clinical presentation of the left foot demonstrating a pedunculated mass overlying the lateral aspect

Upon re-presentation to the podiatry clinic, she reported progressive pain and gradual enlargement of the mass over the lateral fifth metatarsal shaft. The lesion had been concealed beneath a callus and initially lacked overt neoplastic characteristics (Figure 1).

**Figure 2:**
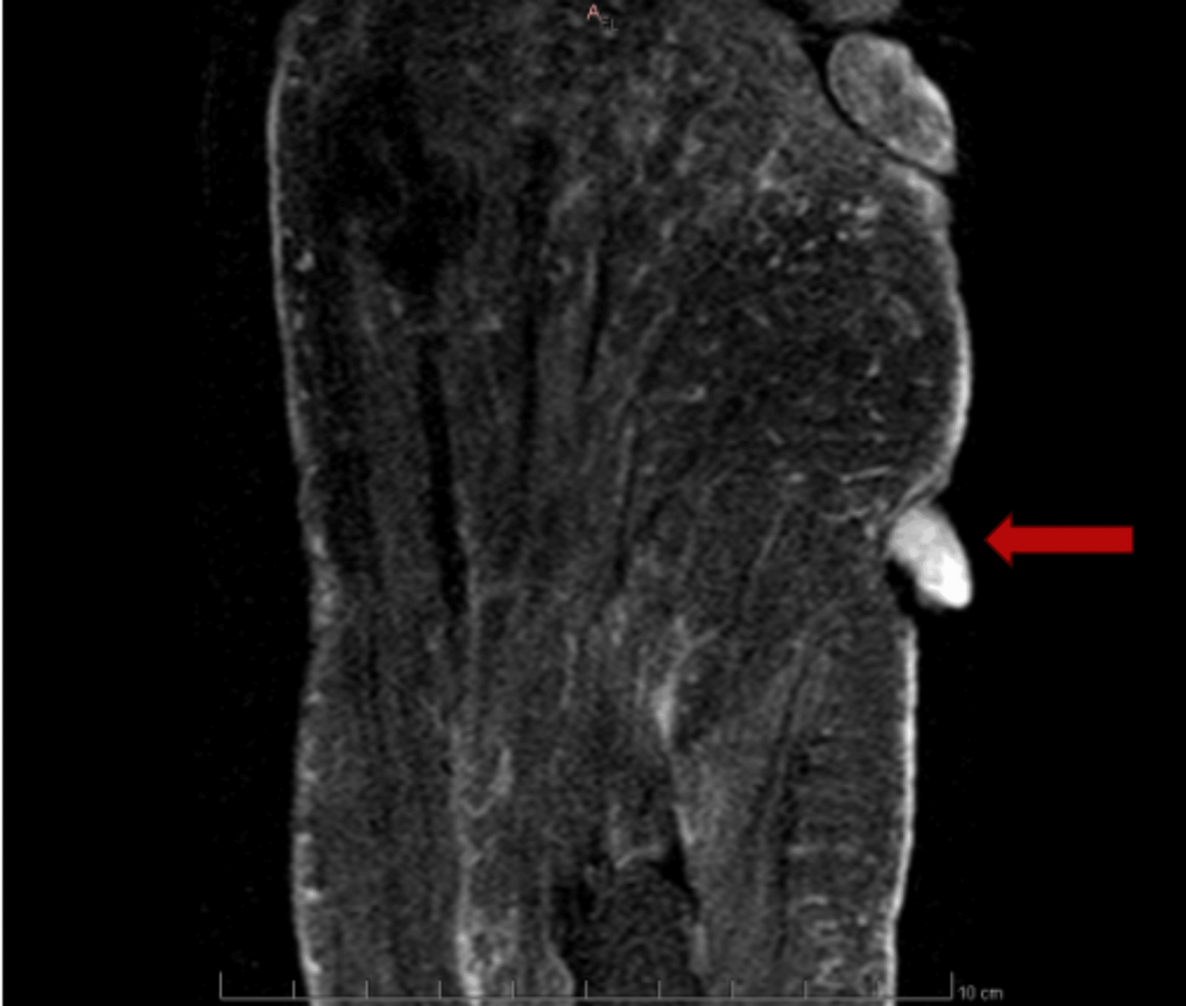
Axial STIR MRI of the left foot demonstrating a hyperintense pedunculated mass (arrow) overlying the lateral aspect of the foot STIR: short tau inversion recovery; MRI: magnetic resonance imaging

Magnetic resonance imaging (MRI) revealed a 1.5 × 1.5 × 1.2 cm pedunculated soft-tissue mass with features concerning for melanoma (Figure 2).

Evaluation of vascular status demonstrated triphasic flow in the external iliac and common femoral arteries and biphasic flow in the profunda femoris and proximal superficial femoral arteries. The superficial femoral artery was occluded from the mid-thigh to the proximal popliteal artery. Blood flow reconstituted at the mid-popliteal artery and continued distally as monophasic flow. The tibioperoneal trunk, posterior tibial, common plantar, and peroneal arteries were occluded. The dorsalis pedis artery demonstrated Category 2 acceleration with a peak systolic velocity of 150 cm/s.

Given the presence of significant peripheral arterial disease, likely exacerbated by long-standing diabetes mellitus, the patient underwent left lower extremity revascularization four months prior to surgical intervention and was subsequently medically optimized for excision.

The patient was brought to the operating room and positioned supine. A pneumatic ankle tourniquet was not utilized. Following intravenous sedation, a total of 15 mL of a 1:1 mixture of 1% lidocaine (plain) and 0.5% bupivacaine (plain) was infiltrated in a V-block around the dorsolateral soft-tissue mass. The left foot was prepped and draped in the usual sterile manner.

An elliptical incision was made over the dorsolateral aspect of the foot using a No. 15 blade and deepened through the subcutaneous tissues. Hemostasis was achieved by identifying, clamping, and cauterizing bleeding vessels. An additional 6 mL of 1% lidocaine (plain) was administered as a field block for intraoperative analgesia. The mass was carefully dissected and excised in its entirety. The wound was irrigated with normal saline and inspected to confirm complete removal of abnormal tissue. Adjacent skin was mobilized to allow tension-free closure. Deep tissues were approximated using 3-0 Vicryl, and the skin was closed with 3-0 nylon sutures. A total of 10 mL of dexamethasone was injected into the operative site.

**Figure 3:**
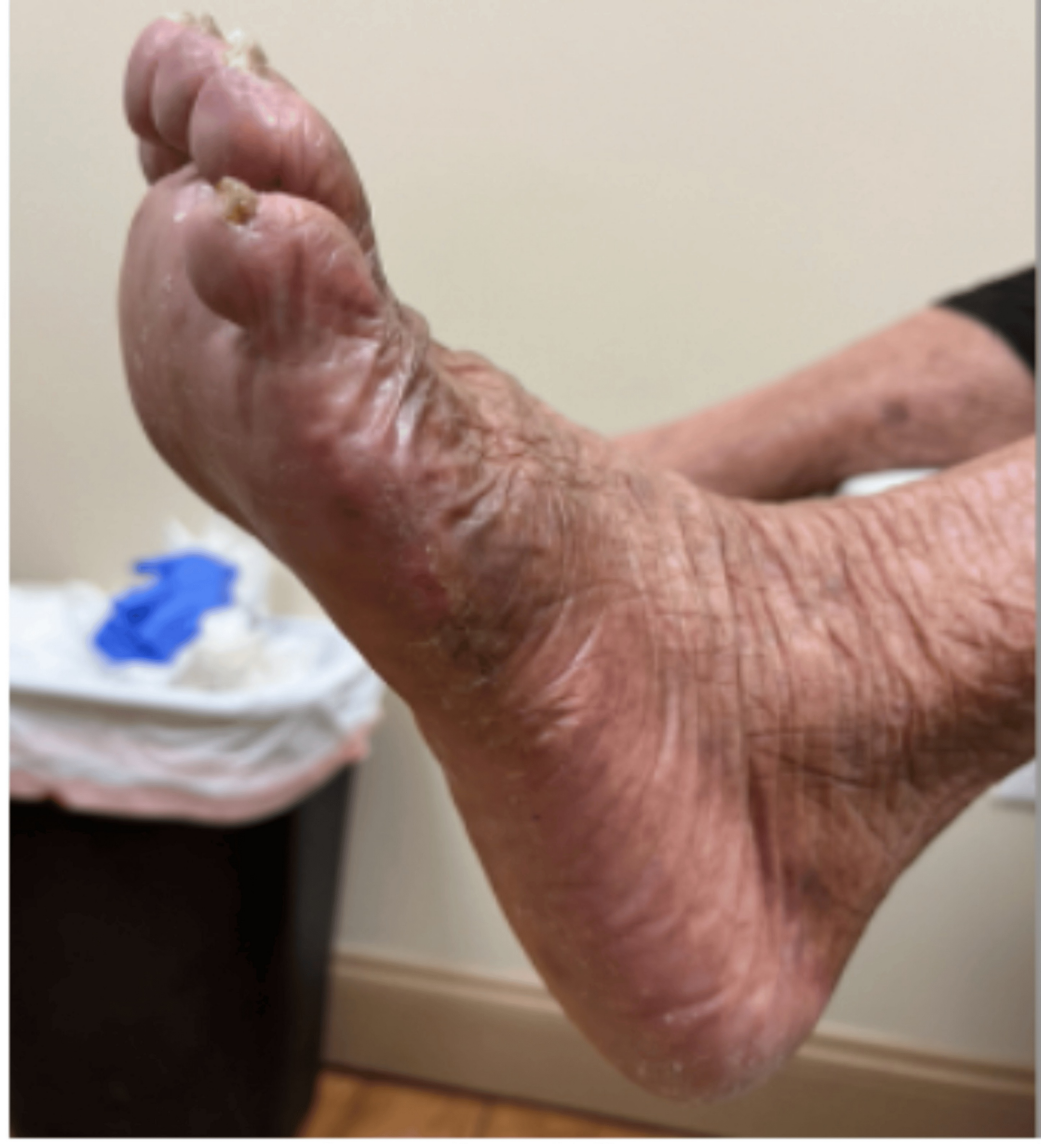
Left foot healed incision site

**Figure 4:**
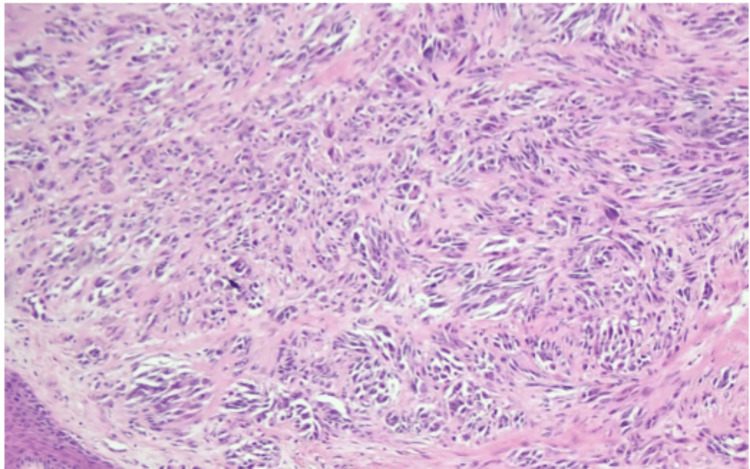
Microphotograph of the excised left foot lesion A) Low-power view demonstrating intersecting sheets and fascicles of spindle cells with marked nuclear atypia (Hematoxylin and Eosin, 100x).

**Figure 5:**
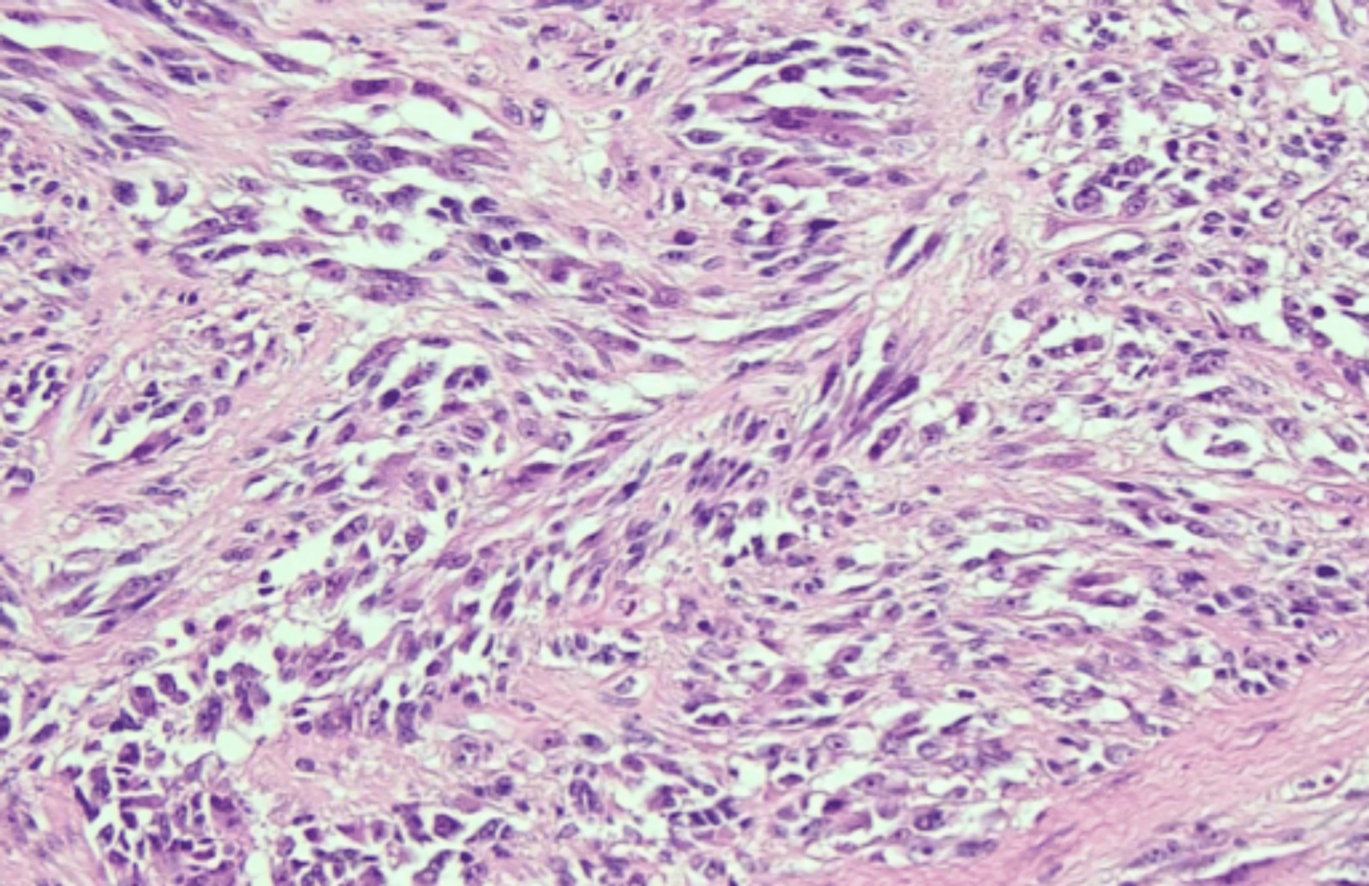
Microphotograph of the excised left foot lesion A) High-power view demonstrating markedly atypical spindle cells with pleomorphic, hyperchromatic nuclei, and minimal mitotic activity (Hematoxylin and Eosin, 400x).

**Figure 6:**
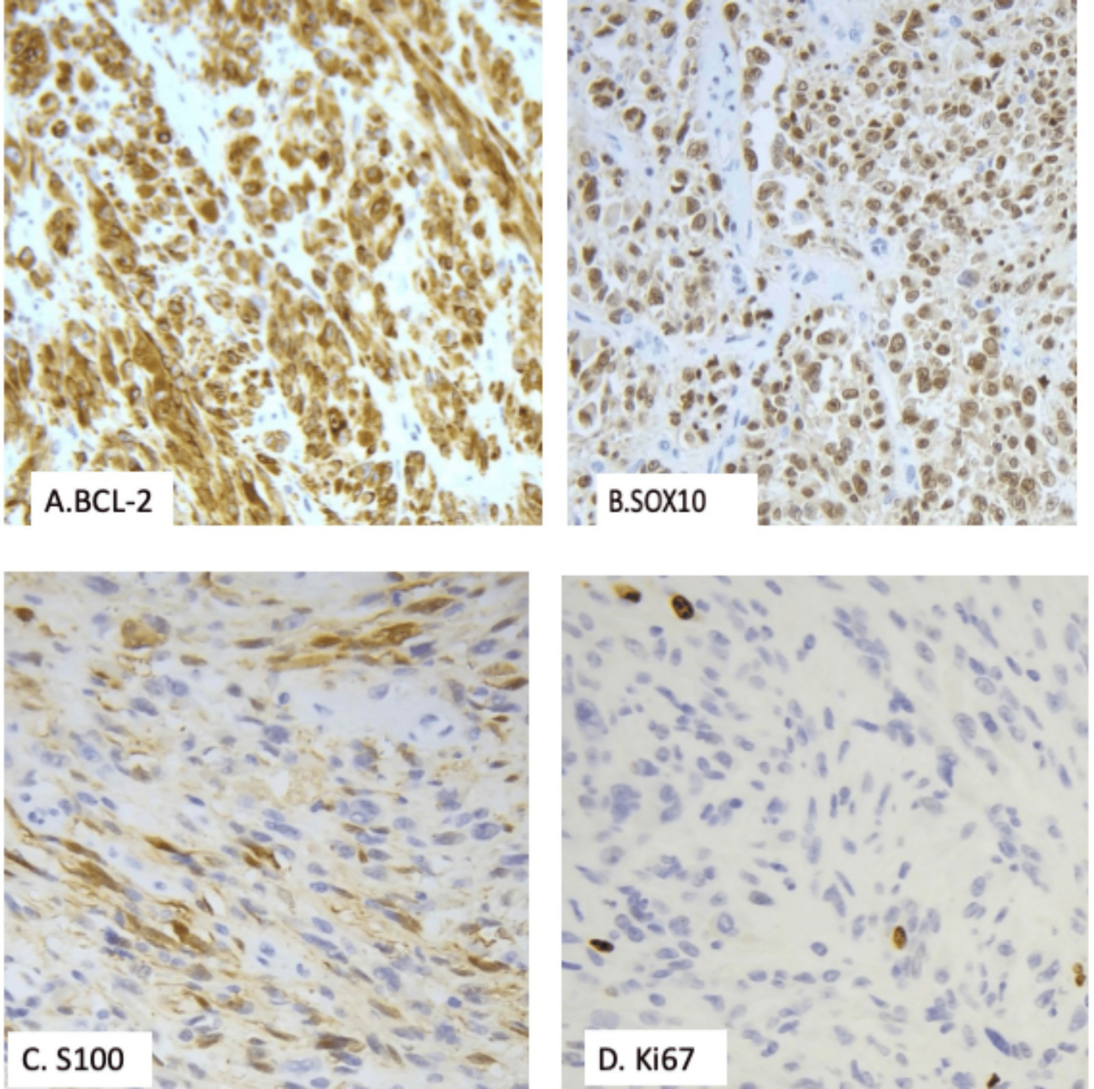
Immunohistochemistry Neoplastic cells demonstrate diffuse, strong positivity for BCL-2 (A, 200x) and diffuse nuclear positivity for SOX10 (B, 200x). Patchy, weak staining for S100 (C, 200x) and a low Ki-67 proliferative index (D, 200x) are also observed.

The specimen, measuring 1.5 × 1.5 × 1.2 cm, was removed without intraoperative or postoperative complications (Figure 3) and submitted for histopathologic evaluation (Figures 4-6).

Histopathologic examination revealed a spindle cell neoplasm composed of intersecting sheets and fascicles with marked nuclear atypia (Figure 4). On higher magnification, pleomorphic, hyperchromatic nuclei were identified without significant mitotic activity (Figure 5). Quantitative histopathologic analysis demonstrated a Breslow thickness of 10.5 mm (pT4a), a low proliferative index (<3% Ki-67), and a mitotic rate of 1 mitosis/mm².

Immunohistochemical analysis demonstrated diffuse, strong positivity for SOX10 and BCL-2, supporting melanocytic differentiation. S100 showed patchy, weak staining, and Ki-67 revealed a low proliferative index (Figure 6).

## Discussion

Foot melanoma is uncommon and frequently presents atypically, contributing to delayed diagnosis and poorer outcomes [7,9,10]. This case illustrates the diagnostic challenges associated with melanoma arising in acral locations, particularly in elderly patients with multiple comorbidities. In older individuals, especially those with diabetes and peripheral neuropathy, cutaneous lesions may be painless, concealed beneath hyperkeratotic tissue, or mistaken for benign entities such as calluses or chronic ulcers [7,9]. This overlap between diabetic foot pathology and acral melanoma has been associated with misdiagnosis and delayed recognition [7,9,10], emphasizing the importance of early biopsy for suspicious or non-healing lesions. Diagnostic delays, exacerbated in recent years, including during the COVID-19 era, have been associated with increased Breslow thickness and more advanced stage at diagnosis [11-15].

The management of melanoma in elderly patients is further complicated by significant comorbid conditions. Cardiovascular disease and malignancy share common risk factors and underlying inflammatory pathways, and patients with peripheral arterial disease have been shown to have an increased risk of incident cancer in large cohort studies, even after adjustment for key confounders such as smoking and diabetes [16]. In this case, severe peripheral arterial disease required revascularization prior to definitive excision, highlighting the importance of multidisciplinary coordination and preoperative optimization in complex geriatric patients.

Histopathologically, this lesion demonstrated features consistent with melanoma NOS, including mixed epithelioid and spindle cell morphology, low mitotic activity, and an atypical immunophenotype. Emerging genomic data suggest that NOS melanomas may represent a biologically distinct subset within the melanoma spectrum rather than a purely exclusionary category [17]. Goldberger et al. demonstrated that NOS melanomas may share favorable histopathologic characteristics with superficial spreading melanoma, yet exhibit paradoxically shorter recurrence-free survival, suggesting unique underlying tumor biology [17].

Despite the tumor’s substantial Breslow thickness (10.5 mm, pT4a), the absence of ulceration and low proliferative index (<3% Ki-67; 1 mitosis/mm²) represent relatively favorable prognostic indicators within an otherwise high-risk lesion. Breslow thickness, ulceration status, and mitotic rate remain the most robust independent predictors of outcome in melanoma [12,14]. While thick melanomas are generally associated with increased metastatic potential, non-ulcerated tumors demonstrate improved survival compared with ulcerated counterparts, underscoring the importance of integrating multiple histopathologic parameters into prognostic assessment [12,14,18].

The immunohistochemical profile in this case underscores the heterogeneity of rare melanoma variants. Diffuse SOX10 positivity with weak or patchy S100 expression, along with negative HMB45 and PRAME, highlights the diagnostic complexity of spindle cell and dedifferentiated melanomas, which may lose conventional melanocytic marker expression. In such scenarios, the use of broad immunohistochemical panels in conjunction with careful morphologic correlation is essential for accurate diagnosis [19-21]. Molecular testing further excluded important mimics, including synovial sarcoma and clear cell sarcoma, strengthening the final diagnosis. The absence of lymphovascular invasion, microsatellitosis, and perineural invasion provides additional prognostic context despite the lesion’s advanced thickness.

This case emphasizes the need for heightened clinical suspicion for atypical lesions of the foot, particularly in elderly or diabetic patients [7,9,10]. Even lesions that appear clinically benign or demonstrate low mitotic activity may represent aggressive melanoma variants [17,19-21]. Early biopsy, comprehensive histopathologic evaluation, and appropriate ancillary testing are critical to ensuring accurate diagnosis and optimal management. Timely recognition of atypical presentations remains essential to improving outcomes in this high-risk and often under-recognized patient population [7,9,10,12,17,19-21].

## Conclusions

This case highlights an unusual presentation of minimally mitotic, amelanotic melanoma arising from the lateral fifth metatarsal shaft in an elderly patient with significant comorbidities. A diagnostic delay of more than three years (>3 years) resulted from its atypical clinical appearance, concealment beneath a callus, and lack of overt malignant features. Despite low proliferative activity, the lesion demonstrated advanced Breslow thickness, underscoring that tumor aggressiveness cannot be reliably inferred from mitotic rate alone.

Clinicians should maintain a high index of suspicion for persistent, enlarging, or atypical acral lesions, particularly in elderly or diabetic patients, and pursue early biopsy and appropriate imaging to facilitate timely diagnosis. This case reinforces the critical importance of early recognition and multidisciplinary management in improving outcomes for these often-overlooked melanoma presentations.
